# Energy Properties and Biomass Yield of Miscanthus x Giganteus Fertilized by Municipal Sewage Sludge

**DOI:** 10.3390/molecules26144371

**Published:** 2021-07-20

**Authors:** Neven Voća, Josip Leto, Tomislav Karažija, Nikola Bilandžija, Anamarija Peter, Hrvoje Kutnjak, Jona Šurić, Milan Poljak

**Affiliations:** 1 Department of Agricultural Technology, Storage and Transport, Faculty of Agriculture, University of Zagreb, Svetošimunska cesta 25, 10000 Zagreb, Croatia; nvoca@agr.hr (N.V.); apeter@agr.hr (A.P.); jsuric@agr.hr (J.Š.); 2 Department of Field Crops, Forage and Grassland, Faculty of Agriculture, University of Zagreb, Svetošimunska cesta 25, 10000 Zagreb, Croatia; hkutnjak@agr.hr; 3 Department of Plant Nutrition, Faculty of Agriculture, University of Zagreb, Svetošimunska cesta 25, 10000 Zagreb, Croatia; tkarazija@agr.hr (T.K.); mpoljak@agr.hr (M.P.); 4 Department of Agricultural Engineering, Faculty of Agriculture, University of Zagreb, Svetošimunska cesta 25, 10000 Zagreb, Croatia; nbilandzija@agr.hr

**Keywords:** sludge utilization, energy crops, waste management, agriculture, non-food production

## Abstract

The application of municipal sewage sludge as fertilizer in the production of non-food energy crops is an environmentally and economically sustainable approach to sewage sludge management. In addition, the application of municipal sewage sludge to energy crops such as *Miscanthus x giganteus* is an alternative form of recycling nutrients and organic material from waste. Municipal sewage sludge is a potential source of heavy metals in the soil, some of which can be removed by growing energy crops that are also remediation agents. Therefore, the objective of the research was to investigate the effect of municipal sewage sludge applied at three different rates of 1.66, 3.22 and 6.44 t/ha on the production of Miscanthus. Based on the analyses conducted on the biomass of Miscanthus fertilized with sludge from the wastewater treatment plant in three fertilization treatments, it can be concluded that the biomass of Miscanthus is a good feedstock for the process of direct combustion. Moreover, the application of the largest amount of municipal sewage sludge during cultivation had no negative effect on the properties of Miscanthus biomass. Moreover, the cellulose and hemicellulose content of Miscanthus is ideal for the production of second-generation liquid biofuels. Fertilizer treatments had no effect on the content of cellulose and lignin, while a significant statistical difference was found for hemicellulose.

## 1. Introduction

The organic matter and nutrients contained in municipal sewage sludge are mostly disposed of in landfills or incinerators, neither of which completely solves the problem [1]. In the European Union, the landfilling of untreated municipal sewage sludge is not allowed according to the legislation 1999/31/EC on the landfilling of waste [2]. In addition, the municipal sewage sludge can be pretreated or combined with other solids prior to its application [3,4]. The treated municipal sewage sludge (stabilized and dehydrated) can be used for landfill cover, but is considered transitional and unsustainable because of the loss of phosphorus and energy that could be used for gas production and cogeneration. For this reason, the use of municipal sewage sludge in agriculture is the most ecologically and economically sound practice because it improves soil properties, provides nutrients to crops and saves valuable water resources, especially in countries with dry climates [5,6]. Estimates show that more than 3.5 million hectares of agricultural land worldwide are irrigated with treated, diluted, partially treated or untreated wastewater [7].

Municipal sewage sludge is a particular inhomogeneous type of waste that must be properly managed. The management of sewage sludge is carried out in different ways in global practice. There is no single strategy or clear guidelines for the management of municipal sewage sludge at the global level. Each country approaches the problem of sewage sludge management in its own way. Even at the EU level, there are currently significant differences in the way municipal sewage sludge is managed from country to country and new methods for its sustainable management are being developed [8]. Municipal sewage sludge is most commonly used in agriculture in the production of non-food crops because it is a rich source of organic matter and macro and micro nutrients. This method of municipal sewage sludge management is very effective as it promotes circular nutrient economy, and can be implemented at relatively low cost as it promotes the recycling of nutrients from organic wastes [6,9,10,11,12,13,14,15]. Municipal sewage sludge is particularly recommended in the production of energy crops with high nutrient requirements, where it can effectively replace mineral fertilizers [6,16]. Therefore, in most countries, agriculture has become the leading route for the final treatment of sludge from municipal wastewater treatment plants. According to Eurostat official data, sludge utilization in agriculture is most widespread in Bulgaria, the Czech Republic, Spain, Cyprus, Hungary, Poland, Portugal, Ireland, Greece, Latvia and Lithuania. In the Netherlands, Switzerland, Belgium, Germany, Austria and Slovenia, sewage sludge incineration is the primary disposal method. Although municipal sewage sludge disposal to landfills is restricted under European directives and almost no longer practiced, it is still predominant in Romania and Italy, and in Malta it is practically the only way of disposing of municipal sewage sludge [17].

According to Eurostat (2020) [17], Croatia currently produces 20,000 tons of municipal sewage sludge per year on a dry basis. On a national level, about 10% of the sludge is used in agriculture; the remaining sludge is mainly disposed of in landfills or exported to other countries. Furthermore, the disposal of municipal sewage sludge in agriculture is defined by strict regulations and guidelines that distinguish between food and non-food production uses. There is a great potential for improvement in terms of the efficient application of sludge on marginal agricultural land, in larger quantities, without risk or possible pollutant input into the environment. The use of municipal sewage sludge on agricultural land as a fertilizer and soil conditioner is the most sustainable type of sludge disposal and soil improvement technique due to its high organic matter and nutrient content, which can improve the chemical, physical and biological properties of soils. In addition to pathogens and volatile organic compounds, municipal sewage sludge can also contain heavy metals that can accumulate and contaminate crops and the food chain. However, the quality and application rate of sludge must be controlled and has been regulated by more or less restrictive rules. Thus, the total amount of municipal sewage sludge applied must be controlled in accordance with the national standards for agricultural use of sewage sludge. Nowadays, in Croatia, the use of municipal sewage sludge produced in municipal wastewater treatment plants is prohibited for agricultural land used for growing food [18]. Since municipal sewage sludge is rich in organic matter and other nutrients, it can be used in a sustainable manner as a fertilizer and soil conditioner for the production of non-food crops [19]. However, as it can introduce various pollutants, such as heavy metals, pathogens, and organic micro pollutants, into the environment and subsequently into the food chain, the use of sewage sludge in energy crop production is also a concern.

The Croatian Ordinance of management of sewage sludge when used in agriculture [20] fully implements the provisions of Council Directive 86/278/EEC, which is applied in all EU countries. The permitted heavy metal content in the dry matter of municipal sewage sludge set by Croatian Ordinance is significantly lower than the levels allowed by the Directive. In the case of cadmium, the Ordinance sets the maximum concentration at 5 mg/kg, while the Directive allows 40 mg/kg of sludge dry matter, which means that the value set in the Ordinance is eight times lower than that allowed by the Directive. The Ordinance sets the limit value for copper three times lower, for nickel and mercury five times lower, for lead more than two times lower and for zinc two times lower. The concentration of heavy metals that may be applied annually to agricultural land (kg/ha/year) is not set in the Ordinance for individual metals as defined by the Directive, but there is a limit of a maximum of 1.66 tons of sludge dry matter per hectare of agricultural land per year.

Despite these limitations, the use of energy crops offers the possibility of using municipal sewage sludge as fertilizer on a large scale. Ideally, the use of municipal sewage sludge would fit seamlessly into energy crop production, which would be freed from the problems with sewage sludge’s health reputation. The market distrusts such a product from a health and environmental safety perspective. Moreover, this method could be used on soils of low quality and in unfavorable climatic conditions that cannot compete with conventional food production [6,21,22]. The cultivation of energy crops will help to meet European criteria for the reduction of greenhouse gas emissions, the production of renewable energy sources and the closure of a whole range of development opportunities and investments in agricultural production. It will contribute to European energy and economic development and increase the security of supply through the use of additional national energy sources. Consequently, the sterile grass Miscanthus (*Miscanthus* x *giganteus* Greed et Deu) is imposed as an ideal energy crop. Specifically, the cultivation of energy crops such as Miscanthus is justified not only by the need to increase the share of renewable energy sources, but it would make it possible to safely and efficiently manage municipal sewage sludge in agriculture [23,24,25]. Miscanthus has a high yield potential within a wide range of environmental conditions and it is characterized by relatively low agronomic requirements. Due to its high resource-use efficiency and tolerance to biotic and abiotic stressors, Miscanthus can be cultivated on marginal land, which plays a minor role in food production [26,27,28,29,30,31,32,33]. It is a crop that improves soil fertility, reduces erosion, has a positive impact on biodiversity and is resistant to diseases and pests. According to Galatsidas et al. (2018) [34], the total area of marginal soils in Europe that can be dedicated to the production of Miscanthus is close to 11 million hectares. At present, the area under Miscanthus is estimated at 19,000 ha in the EU. As shown by many authors [35,36,37,38], Miscanthus can produce a much higher biomass yield after applying a fertilizer, e.g., municipal sewage sludge, which is the source of many valuable nutrients and has a value close to manure, but contains a number of potentially harmful constituents, such as heavy metals or metalloids. The use of municipal sewage sludge could not only increase yields but also positively affect biological and physicochemical properties of the soil profile [6,39,40]. That is why the interest in the use of fertilizer in the cultivation of energy crops such as giant Miscanthus has been studied by many authors [6,37,38,41,42,43,44,45].

The aim of this study was to investigate the effect of the fertilization of municipal sewage sludge on biomass yield and energetic properties of Miscanthus. In addition, the aim was to investigate whether increasing dosages of municipal sewage sludge not only change the properties of Miscanthus but also exert a positive influence on the physical and chemical properties of the soil for biomass production, i.e., changes in soil fertility and the accumulation of heavy metals in soil.

## 2. Results and Discussion

The results show that the changes in soil chemical fertility were statistically similar in the treated and control soils (Table 1). Under experimental conditions, compared with the control treatment, soil pH and available phosphorus decreased due to the increased application rate of municipal sewage sludge, while total nitrogen was almost equal to that of the control soil. At the same time, soil organic carbon content and available potassium increased due to the increase in sewage sludge rate. This low variability in soil fertility could be related to the application method of the municipal sewage sludge, the solubility of the nutrients and the uptake by the cultivated Miscanthus plant. The sludge used had high pH, organic carbon, nitrogen, and phosphorus content, but low potassium content. Municipal sewage sludge applied to the soil surface did not improve soil fertility as seen in many studies with the incorporation of municipal sewage sludge [19,46,47,48,49]. The weak increase in available potassium in the soil follows the application rate of municipal sewage sludge and may be a consequence of its high solubility in sludge. However, the changes in total organic C and total N in the soil could be related to the slow and inefficient process of mineralization of organic matter under the experimental weather conditions. The N and P nutrients in municipal sewage sludge are mainly in organic forms, which cannot be converted into available nutrients until they reach the soil and are gradually mineralized. In addition, the microbes in municipal sewage sludge can have a major impact on the microbiome of the local soil and plant growth, and the impact of temperature on an ecosystem cannot be ignored [50]. Even low concentrations of mineral forms of nitrogen in municipal sewage sludge and higher after mineralization can be lost by volatilization (NH_4_^+^) or leaching (NO_3_^−^), especially if not incorporated into the soil. Sludge applied to low pH soils may decrease soil pH further due to low organic matter mineralization. The changes in heavy metal content in soil due to the application rate of municipal sewage sludge and the maximum limits proposed by the Croatian regulation [18] are presented in Table 2.

An increase in the rate of application of municipal sewage sludge increased the total heavy metal concentration in the soil, apparently due to its composition. The initial content of Cr and Ni in soil exceeded the proposed national limit and increased slightly with increased municipal sewage sludge application rates. However, the increase in the content of Cu, Zn and Pb in the amended soil did not exceed the maximum allowable concentration, with the exception of Cd, the concentration of which is at the edge of the maximum allowable limit for acidic soils [18]. Compared to the control, total Cu and Pb concentrations in the soil increased significantly only due to the application of 3.32 and 1.66 t/ha dry matter of the municipal sewage sludge, respectively. The highest total Cd and Pb soil concentrations were observed only by the 1.66 t/ha municipal sewage sludge application. Regarding Ni, Zn and Cr, no significant differences were observed between the different treatments. Nevertheless, a slight tendency to increase the concentration of these metals in the soli was observed when sewage sludge was applied. The pH of the municipal sewage sludge may influence the application sites by changing the pH of the soil and affecting the uptake of metals by soil and plants.

As Miscanthus reaches maximum plant height, it also reaches maximum biomass yield. The end of the growing season of Miscanthus coincides with the onset of lower temperatures, and full maturation and drying of the crop begins with the onset of the first autumn frost, when the first harvest is made. At the end of the growing season, nutrients are translocated from the above-ground parts of the plant to the rhizomes. In older plantations, this process begins in late summer and early fall. The stems are gradually dried during the winter and early spring when they are ready for harvest (if used as solid fuel). During this period, there is a significant reduction in yield (35–45%) due to the dropping of leaves and upper parts of the stem (broom), but also due to the improvement of the fuel properties of the biomass. Field trials have shown that Miscanthus sinensis hybrids yield up to 25 t/ha of dry matter in northern parts of Europe, Miscanthus x giganteus hybrids up to 38 t/ha of dry matter in central and Southern Europe and certain high-yielding Miscanthus sinensis hybrids up to 41 t/ha of dry matter [51,52,53]. Table 3 shows the values of number of shoots, plant height and percentage of dry matter of Miscanthus harvested in spring 2020.

The mean dry matter yields of the plants varied from 20.42 in the control to 20.81 t/ha dry matter in the treatment with municipal sewage sludge at 3.32 t/ha, depending on the treatment. The study showed that the single use of municipal sewage sludge in the experiment did not significantly increase crop yield compared to the control. Antonkiewicz et al. (2016) [16] found that a dose of 10 t of municipal sewage sludge dry matter increased the yield of Miscanthus by 8% compared to the control. In contrast to our results, Antonkiewicz et al. (2016) [16] found that a dose of 40 and 60 t/ha of municipal sewage sludge dry matter caused a yield decrease of over 17% and 26%, respectively, compared to the control. The average moisture content in the spring harvest period indicates the possibility of storing harvested biomass without a prior process of thermal processing (drying), which directly affects the energy and economic balance of biomass production. Table 3 shows the quality values of Miscanthus biomass that are consistent with or expected to differ from the literature and/or the CEN/TS 14961 standard for solid biofuels [54]. It can be concluded that yield and moisture content in Miscanthus biomass are fundamental parameters for harvesting optimization. In this case, Miscanthus biomass is dried in the field and should be aerated and stored only when necessary. However, it is important to point out that the climatic-meteorological factors of the cultivation microsite are the main criteria for determining the optimal harvest period, which is mainly related to the moisture content in the biomass and the current soil condition. Proximate analysis typically involves determination of dry matter and volatiles, fixed carbon and ash and, together with coke, is the most commonly used method for biomass characterization (Table 4) [55]. Many authors [56,57,58,59,60] considered proximate analysis as the most important chemical properties of biomass for energy production.

Ash is an inorganic part of fuel that remains after biomass is completely burned. The ash content in biomass can vary from 1 to 40%. Since the best-quality biomass has less than 1% ash, it can be noted that the samples of Miscanthus studied had slightly higher ash content, which averaged 1.91% in the study. Since the ash-containing materials have no calorific value, their desirable value should be as low as possible, since biomass with a higher ash content significantly reduces the operating efficiency of the incinerator. Compared to the standard for solid fuels [54], the data show that the studied Miscanthus biomass is much more suitable for combustion processes compared to some other agricultural biomasses. Due to the lower moisture and ash content, the application of Miscanthus for combustion and generation of thermal and electrical energy can be proposed.

The range of volatile matter content in the Miscanthus samples averaged 81.88%, with no statistical differences between the municipal sewage sludge dosages used in fertilizing the field. The value of volatile matter content was similar to the majority of agricultural and forest biomass. In fact, the determination of volatile matter content in biomass is of great importance because it also determines the way biomass is used as fuel. For example, biomass is dried by heating and thermally decomposed, which is manifested by the separation of volatiles from the biomass. This evaporation process (volatilization) takes place until only the non-volatile fraction remains in the fuel. The composition of the volatile fraction of the biomass can be very different. It depends mainly on the composition of the biomass, the temperature of thermal decomposition and the rate of removal of the decomposition products (gasses). It is assumed that at lower temperatures mainly carbon and oxygen compounds are released, while at higher temperatures compounds from hydrogen are released [60].

Coke is a secondary coal formed at higher temperatures. It is a remnant of dry distillation and increases the quality of the fuel. Throughout the study, only coke content behaved in contrast to the other elements studied, i.e., its proportion was affected by the sludge fertilization. Specifically, the highest amount of coke in Miscanthus samples was determined in the control without any sludge fertilization, which was 12.14%. On the other hand, the least amount of coke was found in the two highest dosages of municipal sewage sludge fertilizer. It was found that the dosage of 3.32 kg/ha dry matter of municipal sewage sludge has the lowest coke content in biomass samples by a significant margin—11.44%. However, the obtained values of coke in biomass are favorable for direct combustion and energy production and are within the range found in the literature [55,61,62].

Fixed carbon along with ash is a solid residue after combustion or release of volatiles. An increase in solid carbon increases the calorific value and thus improves the quality of the biomass. Table 4 shows the fixed carbon content in the biomass samples, which averaged 9.78%, which is similar to other agricultural or forestry biomass [57,60,63].

Fuel is a mixture of complex chemical compounds that fall into the category of organic compounds: carbon (C), hydrogen (H), nitrogen (N), and sulfur (S). Fuel consists of a combustible component and ballast (non-combustible components). The combustible components are carbon (C), hydrogen (H), and partially sulfur (S), followed by oxygen (O), which does not burn but allows combustion to occur. Table 5 shows the elemental composition of biomass Miscanthus fertilized with four dosages of municipal sewage sludge, along with the lower (LHV) and higher (HHV) heating values of the biomass Miscanthus samples.

The basic element of biomass is carbon, which makes up 30 to 60% of the dry matter, depending on the ash content, with a higher carbon content increasing the energy value of the biomass. Carbon does not exist freely in biomass, but in organic compounds with oxygen, hydrogen, nitrogen and sulfur. During combustion, the carbon binds to the oxygen and gives off significant amounts of heat energy. During complete combustion, when combustion occurs with a sufficient amount of oxygen, CO_2_ is released. The mean value of carbon content in the samples is 51.65%, which can also be found in the literature [64]. Hydrogen is the basic component of fuel along with carbon, and biomass contains 5 to 6% hydrogen on average, and the higher the hydrogen content, the higher the energy value. Since the hydrogen content of the analyzed samples averaged 6.09%, it can be concluded that the hydrogen content is consistent with the literature, as is the carbon content [63,64,65,66,67,68]. Nitrogen is a macronutrient important for plant growth, and the nitrogen content in biomass varies from 0.2% to more than 1%. During combustion, nitrogen is released in its elemental state and acts as an inert component, meaning that it does not burn or give off heat. It has a negative effect on the activity of the elements with which it is associated and reduces the calorific value. It can produce unwanted nitrogen oxides NO_x_ that pollute the environment. The nitrogen content in the tested samples of Miscanthus averaged 0.18% across all samples, which is a very good indicator because large amounts of nitrogen reduce the heating value of the biomass [59,60,69,70]. Most biomass fuels contain less than 0.2% sulfur, with a few exceptions with higher values of 0.5 to 0.7%. Sulfur in fuel can be combustible and noncombustible. Combustible sulfur is usually bound to organic material or is present in combination with metals. Non-combustible sulfur is stably bound in the form of calcium sulfate, which remains mainly in the ash during and after combustion. Combustion produces sulfur oxides (SO_x_) which pollute the environment. Since biomass has low sulfur content, its combustion does not contribute significantly to sulfur emission. In the samples studied, the average sulfur value in biomass was 0.08%, which is an ideal value for this type of fuel [55,71,72,73]. In contrast, the presence of oxygen in fuel is undesirable because oxygen does not burn but participates in combustion. It usually occurs in compounds with other elements and makes them incombustible, so it reduces the effect of the fuel elements with which it is in contact, resulting in a reduction in the calorific value of the fuel. The oxygen content in the samples averaged 42.00%. The tested samples of Miscanthus have a slightly higher oxygen content, as the average oxygen content in biomass is between 30% and 40%, but it is still within the literature data [57,61,62,74].

One of the basic indicators of a substance’s usefulness as a fuel is its calorific value. Heating values vary depending on the type and composition of the biomass as well as the water content. As the water content in a fuel increases, its calorific value decreases to a greater or lesser extent. The basic definition of the calorific value of a fuel is the amount of heat released by the complete combustion of a unit quantity of fuel when the flue gasses are cooled to the temperature at which the fuel and air are introduced into the combustion chamber. The calorific value of a fuel is the amount of heat produced by the complete combustion of a unit quantity of fuel. The higher heating value (HHV) is the quantity of heat produced by the complete combustion of a unit quantity, the flue gasses being cooled to a temperature of 25 °C and moisture being separated from them as condensate. The lower heating value (LHV) is the quantity of heat produced by the complete combustion of a unit quantity of fuel, the flue gasses being cooled to a temperature of 25 °C and the moisture contained in them remaining in a vaporous state and the heat of the condensate remaining unused [58,59,60]. Table 5 shows the lower and upper heating value of Miscanthus grown in four stages of sludge fertilization. The average upper heating value of Miscanthus biomass is 17.78 MJ/kg and the average lower sample value is 16.45 MJ/kg, which is also within the limits for agricultural biomass [75]. However, it is important to note that the fertilizer treatments had no influence on the heating values of the studied Miscanthus.

The statistical analysis of the previous two tables, in which the ultimate and elemental analysis of the biomass were analyzed, showed that the fertilizer treatments had no negative effect on the change in the composition of the Miscanthus biomass. It was concluded that fertilization had no negative impact on the investigated fuel parameters of Miscanthus energy values, except for the coke content, where there were significant changes. However, according to the literature, these values are also within the recommended values, which range from 9.5% to 15.8% [61,62,63,64,76].

Biomass contains varying amounts of cellulose, hemicellulose, and lignin, as well as small amounts of other components (lipids, proteins, simple sugars, and starch). The ratio of cellulose to lignin is one of the more important factors in determining the suitability of a particular plant species for energy production. It is desirable that there is a lower proportion of cellulose and hemicellulose in biomass in the combustion process, i.e., biomass with higher lignin content is more suitable for direct combustion processes. Table 6 shows the values of cellulose, hemicellulose and lignin content in biomass samples of Miscanthus grown in four different sludge fertilization treatments. The cellulose content averaged 50.45%, which is within the range of literature values for agricultural and forest biomass. On the other hand, the lignin content is slightly lower compared to other plants, while the content of hemicellulose is as high as that of cellulose. It can be concluded that the lignocellulosic composition of Miscanthus is also very good for conversion into second-generation bioethanol [77,78,79]. In addition, the results show that the hemicellulose content was significantly affected by the sludge treatment, which showed significant differences in the treatment with 3.32 t/ha of sludge dry matter compared to the control.

Micro and macro elements immediately after the combustion process form the composition of the resulting ash and some of them can cause a number of serious problems in the combustion chambers, causing slag, corrosion and dirt. The extent of these problems is closely related to the type of biomass used, i.e., the percentage of each element in it. For example, the ratio of potassium to calcium should be considered when assessing biomass quality because of its significant influence on the occurrence of slag. It has been shown that biomass with high calcium and low potassium content is more suitable for combustion and energy production. Potassium and sodium, in combination with sulfur, are involved in the formation of corrosion. These elements partially evaporate during combustion and form alkaline chlorides that condense on the surfaces of the heat exchanger and react with the flue gasses to form sulfates. Consequently, the fuel is of better quality when a lower proportion of potassium and sodium is present. In general, agricultural biomass contains much lower concentrations of heavy metals than forest biomass. This can be explained by the long rotation time of trees, which improves the phytoaccumulation of heavy metals, and by lower pH values of forest soils, which influence the increase in the solubility of heavy metals [60,79,80]. Of the micro and macro elements, the following proportions were studied: sodium (Na), calcium (Ca), potassium (K), magnesium (Mg), manganese (Mn), iron (Fe), zinc (Zn), copper (Cu), chromium (Cr), lead (Pb), nickel (Ni) and cobalt (Co) (Table 7 and Table 8).

Sodium, in combination with chlorine and sulfur, is involved in the formation of corrosion and partially evaporates on combustion, forming sulfates and releasing chlorine. Therefore, the lower the sodium content, the better the fuel quality. The lowest sodium content was found in the control treatment without sludge fertilization, while the highest was found in the fertilization treatment with the highest amount of sludge, although these differences were not statistically significant. Calcium reacts with potassium and silicon and affects the appearance of slag in furnaces. Its increased content contributes to a lower possibility of slag appearance, but also lowers the melting point. In the biomass of Miscanthus studied, the calcium content increased with the amount of slurry used in cultivation, and these differences are significant. The highest amount of calcium was found in the biomass fertilized with the highest amount of municipal sewage sludge and the lowest in the control-without the use of municipal sewage sludge in the cultivation of Miscanthus. Potassium, in combination with chlorine and sulfur, is involved in the formation of corrosion and partially evaporates during combustion, forming sulfates and releasing chlorine. Therefore, the lower the potassium content, the better the fuel. In our case, the highest amount of potassium was found in biomass fertilized with 1.66 t/ha dry matter sludge, but the lowest amount of potassium in biomass was found in samples fertilized with 3.32 t/ha. Magnesium, as an alkaline element, readily forms a mixture of two or more solid phases, lowering the melting point and usually increasing the melting temperature of the ash. As for sodium, no statistical regularity was found for magnesium in relation to the importance of municipal sewage sludge application.

Iron, copper, manganese, and zinc, as heavy metals, are an undesirable component in the biomass composition, so their lowest possible concentration is desirable. The application of municipal sewage sludge had no significant effect on all the microelements studied as well as on heavy metals except manganese, the highest amount of which was found in the samples fertilized with 3.32 t/ha of municipal sewage sludge. In no case, except for manganese, were significant differences found between biomass samples fertilized with different amounts of municipal sewage sludge. In addition, the content of heavy metals nickel, lead, cadmium, chromium and cobalt was not found in any sample of Miscanthus biomass, because their concentration was very low, i.e., it was below the sensitivity limit (0.25 mg/kg) of the device used in this study.

## 3. Materials and Methods

### 3.1. Scheme and Conditions of the Field Experiment

**Figure 1:**
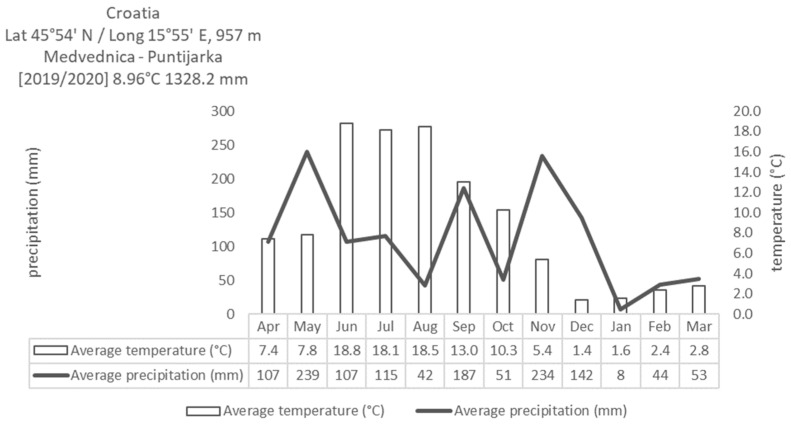
Meteorological data of precipitation and average daily temperature during the growing season of Miscanthus.

The Miscanthus (*Miscanthus* x *giganteus* Greed et Deu) plantation was established in April 2011 in the Center for grassland production (45°92′71″ N, 15°97′36″ S, elevation 650 m). The Centre comprises around 30 ha of total grassland experimental fields, and it is a part of the Faculty of Agriculture University of Zagreb. Miscanthus rhizomes were planted in April 2011 at a density of 1 m^2^, with 1 m inter row spacing. The experiment was organized as a randomized split-splot design with two treatment factors: municipal sewage sludge application and harvest date on plots with a harvesting area of 1500 m^2^ with three replicates. Four different sludge rates (based on 25% dry matter sludge) were used: 0 kg/ha (control), 1.66 t/ha (6.64 t fresh sludge), 3.32 (13.28 t fresh sludge) and 6.64 t/ha (25.56 t fresh sludge) dry matter per year. Municipal sewage sludge was used once; it was mixed with the surface soil layer at surphace in March 2019. Harvesting took place in March 2020, before the start of the new growing season. Monthly average temperature and precipitation at the experimental field of Miscanthus during the growing season is shown in Figure 1.

### 3.2. Soil and Municipal Sewage Sludge

A field trial was conducted with varying amounts of municipal sewage sludge applied once without additional fertilization with commercial fertilizer. The trials are laid out in a block design with four blocks, with all treatments included in each block. All treatments have four replicates. The treatments include completely untreated control plots to which no municipal sewage sludge or mineral fertilizer was added. Municipal sewage sludge at 1.66, 3.32, and 6.64 kg/ha dry matter was applied once to the surface of the Miscanthus tilled soil.

**Figure 2:**
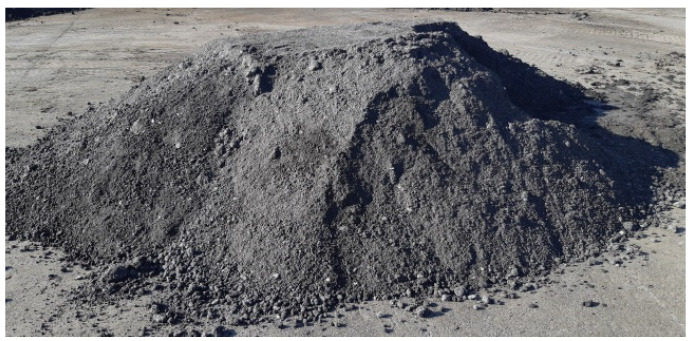
Stabilized municipal sewage sludge used in the investigation.

The municipal sewage sludge used for the experiment was collected from the municipal sewage treatment plant of the city of Zagreb and its quality met the Croatian State Standard for Agricultural Application of Municipal Sewage Sludge (Figure 2) [20]. The basic characteristics of the soil and municipal sewage sludge are presented in Table 9.

Table 9 shows the chemical composition of the municipal sewage sludge (dry weight) used in the experiment, the pH was determined with the glass electrode using a soil-water suspension of 1:10 (*w/v*), dry matter was determined by the gravimetric method (550 °C), the electrical conductivity was determined in the conductive meter (soil/water ratio, 1:10), total nitrogen was determined by the Kjeldahl method and concentrations of total phosphorus (P), potassium (K) and heavy metals Cd, Cr, Cu, Ni, Pb and Zn were measured in aqua regia extraction at AAS using the graphite and hydride technique (SOLAR AA Spectrometer M Series, Thermo Scientific, 2008 using Graphite Furnace and Cold Vapor System).

Soil analysis: soil samples were taken before the application of municipal sewage sludge to determine the soil properties according to the Croatian State Standard for Agricultural Application of Municipal Sewage Sludge [20]. Soil core samples (0–30 cm) were collected from each treatment on 30 March 2019 after harvesting the Miscanthus plant to determine changes in soil properties. Soil organic carbon was determined using the K_2_Cr_2_O_7_ method of external heating. Soil pH was determined using a pH meter (soil to water ratio 5:1). Total nitrogen (N) in soil was determined by the Kjeldahl method. Available phosphorus (P) and potassium (K) were measured by the AL method—extraction with ammonium lactate-acetic acid at a ratio of 1:20 (*m*/*v*). Total heavy metals in soil were measured in aqua regia extraction at AAS using graphite and hydride technique (SOLAR AA Spectrometer M Series, Thermo Scientific, 2008 using Graphite Furnace and Cold Vapor System).

### 3.3. The Determination of Drymatter Yield and the Biomass Characterization

At the end of the growing season in March 2020, the following characteristics of Miscanthus are determined.

- Plant height (m) (10 randomly selected plants per base plot, measured from the soil surface and the top of the flower).
- Number of shoots per m^2^ (10 randomly selected sites of 1 m^2^ per base plot by manual counting of shoots).
- Dry matter yield (t/ha) by cutting the plants on the calculation subplots: 2 × 5 m (Miscanthus height 5 cm above the ground, weighing the harvested mass on a digital scale, drying subsamples of about 1000 g of chopped mass 48 h at 60 °C in a dryer with a fan, reweighing the dried mass and recalculating t/ha. The yield of dry matter is determined in the spring harvest period (end of March/beginning of April).
- Dry matter content (%) is determined according to the following formula: Mass of dried sample after 48 h at 60 °C × 100/mass of fresh sample before drying.
- Moisture content (%) (determined according to the formula: 100-dry matter content).

Analytical testing of samples of Miscanthus biomass was performed in the laboratory of the Faculty of Agriculture University of Zagreb. All samples were immediately transported to the laboratory and dried in the laboratory dryer (model 30-1060, Memmert, Schwabach, Germany) to allow for the comparison of samples under identical operating conditions. After drying, the samples were ground in a laboratory mill (IKA Analysentechnik GmbH, Staufen, Germany). Each sample was analyzed three times to ensure reproducibility of the analyses.

Miscanthus biomass samples were characterized by proximate analysis according to standard methods: Moisture content [81] (CEN/TS 14774-2:2009) in a laboratory furnace (INKO ST-40, Zagreb, Croatia), while ash [82] (EN 18122:2015), solid carbon (by difference), coke and volatile matter [83] (CEN/TS 15148:2009) were determined using a muffle furnace (Nabertherm GmbH, Nabertherm Controller B170, Lilienthal, Germany). Total carbon, hydrogen, nitrogen and sulfur were determined simultaneously by the dry combustion method in a Vario Macro CHNS analyzer (Elementar Analysensysteme GmbH, Langenselbold, Germany) according to the protocols for the determination of carbon, hydrogen and nitrogen [84] (EN16948:2015) and sulphur [85] (EN 16994:2015). The oxygen content was also calculated by difference.

The calorific value was determined according to the ISO method [86] (EN 14918:2010) using an IKA C200 oxygen bomb calorimeter (IKA Analysentechnik GmbH, Staufen, Germany). A 0.5 g sample was weighed into a quartz crucible and placed in the calorimeter for combustion. The higher heating value was determined after combustion using IKA C200 software. The calorific value is expressed in MJ/kg on dry basis. 

The determination of cellulose, hemicellulose and lignin content was performed according to the methodology of Van Soest and Robertson [87] in an ANKOM 2000 analyzer (Macedon, New York, USA).

Analysis of micro and macro elements was carried out by atomic absorption spectroscopy (Perkin Elmer, AAnalyst 400 Waltham, Massachusetts; USA ); samples were previously digested in a microwave sample preparation oven (ETHOS D Milestone, UK) according to standard methods [88] (RN EN ISO 16967, 2015; [89] HRN EN 16968, 2015). The contents of the following micro elements were analyzed: iron (Fe), zinc (Zn), copper (Cu), manganese (Mn), chromium (Cr), lead (Pb), nickel (Ni), cadmium (Cd) and cobalt (Co). Macro elements in the content analysis included sodium (Na), potassium (K), calcium (Ca) and magnesium (Mg).

Statistical analysis of the study results was carried out using Statistica package version 10 PL [90]. The significance of differences between means was tested using Tukey’s test at significance level α ≤ 0.05. For selected (parameter) relationships, the value of Pearson’s linear correlation index (r) was calculated at a significance level of *p* 0.05. A maximum degree of scatter of 5% between measurements in chemical analysis was assumed in the study.

## 4. Conclusions

The required increase in energy production from biomass, a fundamental source of renewable energy, requires the use of new, highly efficient and cost-effective crop production technologies that do not compete with food production. Certified municipal sewage sludge can be used in the production of biomass for energy on poor soils where crops for food production are not grown. Its use reduces the cost of energy crop production, improves soil properties and biological life, and significantly increases biomass yields.

The use of municipal sewage sludge during the cultivation of Miscanthus did not increase the content of heavy metals in the soil, nor in the biomass of Miscanthus, but it improves the quality of the properties of the soil. A low variability of the ratios between micro and macro elements in Miscanthus biomass was observed. The levels of micro- and macro elements and heavy metals in the biomass were optimal, regardless of the dosage of fertilizer sludge used in the cultivation of Miscanthus. On the other hand, higher doses of municipal sewage sludge tended to result in a slightly higher yield of collected biomass, but this difference was not significant. 

From the above facts, it can be concluded that the application of treated municipal sewage sludge in non-food crops is a safe and promising way of managing waste, especially for countries such as Croatia. On the other hand, there is great potential for the use of municipal sewage sludge as a source of organic matter for the reasonable production of other energy crops.

## Figures and Tables

**Table 1:** Chemical properties of soils amended with municipal sewage sludge in single application rates and cultivated with Miscanthus.

Sludge Treatment	pH H_2_O	pH KCl	Humus (g/kg)	Organic Carbon (g/kg)	Total N (g/kg)	P_2_O_5_ (mg/kg)	K_2_O (mg/kg)
Control	5.81	4.45	21.8	12.6	1.9	84.8	241
1.66 t/ha	5.57	4.10	24.3	14.1	1.8	74.5	213
3.32 t/ha	5.87	4.56	24.0	13.9	1.9	72.3	279
6.64 t/ha	5.76	4.47	22.4	13.0	1.9	56.5	305
Average	5.75 ± 0.11	4.39 ± 0.17	23.1 ± 1.06	13.4 ± 0.61	1.87 ± 0.03	72.0 ± 10.1	259.5 ± 35.1
Significance	ns	ns	ns	ns	ns	ns	ns

Values are the mean ± SD of three replicates. Values without letters are not significantly different at *p* ≤ 0.05 by Tukey’s HSD test. Significance: ns, non-significant.

**Table 2:** Total concentration of heavy metals in soils treated with municipal sewage sludge at a single application rate and planted with Miscanthus.

Sludge Treatment	Cd (mg/kg)	Cr (mg/kg)	Cu (mg/kg)	Ni (mg/kg)	Pb (mg/kg)	Zn (mg/kg)
Control	0.56	65.70	25.73 ^b^	43.53	24.18 ^b^	77.85
1.66 t/ha	1.01	65.58	26.20 ^b^	45.63	31.93 ^a^	78.05
3.32 t/ha	0.69	71.60	28.25 ^a^	48.88	29.70 ^ab^	80.35
6.64 t/ha	0.61	66.60	25.78 ^b^	44.70	26.98 ^ab^	79.63
Average	0.71 ± 0.17	67.36 ± 2.47	26.48 ± 1.03	45.68 ± 1.98	28.19 ± 2.90	78.96 ± 1.05
Significance	ns	ns	***	ns	ns	ns
Max. permissible concentration (pHKCl < 5.0)	1.0	40	60	30	50	60

Values are the mean ± SD of three replicates. Different letters in each row indicate significant difference at *p* ≤ 0.05 by Tukey’s HSD test. Values without letters are not significantly different. Significance: *** *p* < 0.001; ns, non-significant.

**Table 3:** Number of shoots, plant height, dry matter yield and dry matter of Miscanthus fertilized with different dosages of municipal sewage sludge.

Sludge Treatment	Number of Shoots/m^2^	Plant Height (m)	Dry Matter Yield (t/ha)	Dry Matter (%)
Control	79.42	2.80	20.42	85.08
1.66 t/ha	81.58	2.69	20.49	85.04
3.32 t/ha	80.28	2.67	20.81	84.97
6.64 t/ha	79.15	2.76	20.47	85.50
Average	80.11 ± 10.09	2.73 ± 0.56	20.55 ± 9.17	85.15 ± 5.68
Significance	ns	ns	ns	ns

Values are the mean ± SD of three replicates. Values without letters are not significantly different at *p* ≤ 0.05 by Tukey’s HSD test. Significance: ns, non-significant.

**Table 4:** Proximate analysis of Miscanthus biomass fertilized with different dosages of municipal sewage sludge.

Sludge Treatment	Ash (%)	Coke (%)	Fixed Carbon (%)	Volatile Matters (%)
Control	2.01	12.14 ^b^	10.14	81.46
1.66 t/ha	1.84	11.70 ^ab^	9.86	81.83
3.32 t/ha	1.95	11.44 ^a^	9.49	82.10
6.64 t/ha	1.84	11.46 ^a^	9.62	82.12
Average	1.91 ± 0.47	11.69 ± 0.94	9.78 ± 1.01	81.88 ± 1.97
Significance	ns	ns	ns	ns

Values are the mean ± SD of three replicates. Different letters in each row indicate significant difference at *p* ≤ 0.05 by Tukey’s HSD test. Values without letters are not significantly different. Significance: ns, non-significant.

**Table 5:** Elemental analysis of Miscanthus biomass fertilized with different dosages of municipal sewage sludge.

Sludge Treatment	C (%)	H (%)	N (%)	S (%)	O (%)	HHV (MJ/kg)	LHV (MJ/kg)
Control	51.52	6.09	0.18	0.07	42.14	17.64	16.31
1.66 t/ha	51.74	6.09	0.16	0.08	41.93	17.88	16.55
3.32 t/ha	51.75	6.10	0.21	0.08	41.86	17.86	16.53
6.64 t/ha	51.58	6.09	0.16	0.09	42.08	17.72	16.39
Average	51.65 ± 1.12	6.09 ± 0.07	0.18 ± 0.12	0.08 ± 0.02	42.00 ± 1.09	17.78 ± 0.36	16.45 ± 0.36
Significance	ns	ns	ns	ns	ns	ns	ns

Values are the mean ± SD of three replicates. Values without letters are not significantly different at *p* ≤ 0.05 by Tukey’s HSD test. Significance: ns, non-significant.

**Table 6:** Lignocellulose, hemicellulose and lignin content of biomass of Miscanthus fertilized with municipal sewage sludge.

Sludge Treatment	Lignocelulose (%)	Hemicelulose (%)	Lignin (%)
Control	50.20	24.87 ^b^	13.89
1.66 t/ha	50.79	23.82 ^ab^	13.71
3.32 t/ha	50.44	23.25 ^a^	13.84
6.64 t/ha	50.40	23.84 ^ab^	13.78
Average	50.45 ± 3.31	23.95 ± 2.01	13.80 ± 1.12
Significance	ns	***	ns

Values are the mean ± SD of three replicates. Different letters in each row indicate significant difference at *p* ≤ 0.05 by Tukey’s HSD test. Values without letters are not significantly different. Significance: *** *p* < 0.001; ns, non-significant.

**Table 7:** Macro element content of Miscanthus biomass fertilized with municipal sewage sludge.

Sludge Treatment	Na (mg/kg)	Ca (mg/kg)	K (mg/kg)	Mg (mg/kg)	P (mg/kg)
Control	46.61	683.46 ^a^	653.09 ^b^	64.58	748.85 ^a^
1.66 t/ha	49.38	731.53 ^ab^	749.72 ^c^	69.24	710.81 ^a^
3.32 t/ha	49.51	768.26 ^ab^	771.77 ^c^	63.11	1077.38 ^b^
6.64 t/ha	50.57	779.95 ^b^	573.29 ^a^	68.55	693.59 ^a^
Average	49.02 ± 3.55	740.80 ± 52.88	686.96 ± 113.88	66.37 ± 14.95	807.66 ± 201.32
Significance	ns	***	***	ns	***

Values are the mean ± SD of three replicates. Different letters in each row indicate significant difference at *p* ≤ 0.05 by Tukey’s HSD test. Values without letters are not significantly different. Significance: *** *p* < 0.001; ns, non-significant.

**Table 8:** Micro element content of Miscanthus biomass fertilized with municipal sewage sludge.

Sludge Treatment	Fe (mg/kg)	Cu (mg/kg)	Mn (mg/kg)	Zn (mg/kg)
Control	100.77	6.23	64.58	28.23
1.66 t/ha	158.13	6.89	69.24	24.06
3.32 t/ha	105.92	6.87	63.11	23.24
6.64 t/ha	86.65	6.88	68.55	25.72
Average	112.87 ± 23.75	6.71 ± 1.57	66.37 ± 14.95	25.31 ± 1.13
Significance	ns	ns	ns	ns

Values are the mean ± SD of three replicates. Values without letters are not significantly different at *p* ≤ 0.05 by Tukey’s HSD test. Significance: ns, non-significant.

**Table 9:** Basic properties of municipal sewage sludge and soil used in this study and permissible heavy metal contents according to the Croatian legislation for the use of municipal sewage sludge in agriculture.

Items	Unit	MSS	Soil	Permitted Total Heavy Metals Content [20]	
				MSS	Soil (for pHKCl 5.0 to 5.9)
pH	-	12.05	5.10	-	-
EC	mS/cm	6.87	-	-	-
Dry matter	%	30.28	-	-	-
C organic	%	28.64	1.21	-	-
N total	%	4.03	0.19	-	-
Ca total	%	14.56	-	-	-
P_2_O_5_ total	%	3.89	-	-	-
K_2_O total	%	0.65	-	-	-
P_2_O_5_ available	mg/kg	-	73	-	-
K_2_O available	mg/kg	-	208	-	-
Cd	mg/kg	0.72	0.60	5	0.5
Cr	mg/kg	77.22	76.0	500	50
Cu	mg/kg	285.59	28.8	600	40
Ni	mg/kg	43.13	64.4	80	30
Pb	mg/kg	66.56	22.7	50	50
Zn	mg/kg	22.66	87.8	2000	100

MSS—municipal sewage sludge.

## Data Availability

Data generated during the study can be obtained by the authors of this study.

## Sample Availability

Samples are not available from the authors.
